# Pediatric computed tomography scan and subsequent risk of malignancy: a nationwide population-based cohort study in Korea using National Cancer Institute dosimetry system for computed tomography (NCICT)

**DOI:** 10.1186/s12916-025-04235-3

**Published:** 2025-07-01

**Authors:** Sangsoo Han, Jaewan Soh, Sangun Nah, Kyungdo Han, Jin-Hyung Jung, Jiwon Park, YoonJoong Hwang, Choonsik Lee, Jae-Young Hong

**Affiliations:** 1 Present Address: Department of Emergency Medicine, Soonchunhyang University Bucheon Hospital, Bucheon, South Korea; 2 Present Address: Department of Orthopaedic Surgery, Hanyang University Guri Hospital, Guri, South Korea; 3 Department of Statistics and Actuarial Science, Soongsil University, Seoul, Korea; 4 Department of Orthopedics, Division of Spinal Surgery, College of Medicine, Korea University, 123, Jeokgeum-Ro, Danwon-Gu, Gyeonggi-Do Ansan-Si, 15355 South Korea; 5 Radiation Epidemiology Branch, Division of Cancer Epidemiology & Genetics, National Cancer Institute, Rockville Pike, USA

**Keywords:** Computed tomography, Neoplasms, Cohort studies, Pediatrics

## Abstract

**Background:**

Computed tomography (CT) has advanced medical diagnostics by offering detailed anatomical imaging, but its use in children raises concerns due to higher radiation doses and increased vulnerability. This study enhances prior research by using organ-specific radiation dose calculations for a more precise cancer risk assessment, investigating the associations between pediatric cancers and radiation doses in a large population cohort.

**Methods:**

This nationwide cohort study analyzed National Health Insurance Service claims data from 2007 to 2015 with a focus on individuals < 20 years of age who underwent CT scans. We used the International Classification of Diseases Tenth Revision codes to identify an exposed cohort and excluded subjects with congenital anomalies or previous cancer diagnoses. The study had a 2-year lag period to minimize selection bias and reverse causation effects. We calculated the exposed organ dose for each organ during each CT scan using the national CT dose survey data and the National Cancer Institute for Computed Tomography (NCICT) dose calculator. Cox proportional hazards regression was used to estimate hazard ratios (HRs) and 95% confidence intervals (CIs) for cancer incidence according to organ-specific radiation dose.

**Results:**

From 2007 to 2015, 1,540,633 children underwent CT scans, with 1,380,896 being included in the final analysis. A significant dose–response relationship was observed: for every one standard deviation increase in organ-specific radiation dose, the overall cancer risk increased (HR 1.155, 95% CI: 1.139–1.171). Among solid malignancies, associations were observed for urinary cancer (HR 1.385, 95% CI: 1.291–1.486), thyroid cancer (HR 1.248, 95% CI: 1.218–1.278), brain cancer (HR 1.201, 95% CI: 1.177–1.225), and digestive system cancer (HR 1.285, 95% CI: 1.240–1.331). Hematologic malignancies, including leukemia (HR 1.074, 95% CI: 1.053–1.100) and other myeloid tumors (HR 1.087, 95% CI: 1.062–1.112), also showed increased risks.

**Conclusions:**

This study revealed a significant relationship between increased radiation doses during CT and the potential risk of various cancers in pediatric patients. Although CT is an invaluable diagnostic tool for which the risks are not high using the current diagnostic doses, a risk/benefit analysis is appropriate, especially for children.

**Supplementary Information:**

The online version contains supplementary material available at 10.1186/s12916-025-04235-3.

## Background

Since its introduction in the early 1970s, computed tomography (CT) has revolutionized medical diagnostics, providing unparalleled insight into the intricate structures of the human body [1]. Despite these remarkable advantages, CT scans deliver higher doses of radiation compared to other diagnostic radiological procedures [2]. Consequently, the increasing use of CT to evaluate pediatric patients has become a major concern in the medical and health communities [3, 4]. Children, given their rapid cell division, developing tissues, and longer expected lifespans post-CT, are particularly sensitive to the effects of radiation, thus amplifying the long-term risks and rendering radiation protection a critical issue in pediatric medicine [5].

Recent studies have emphasized that exposure of children to ionizing radiation can lead to serious complications, including malignant tumors [6–8]. In Australia, the incidence rate (IR) of all cancers in individuals aged 0–19 years exposed to CT was reported to be 9.38 cases per 100,000 person-years (PY) [9]. Further, Pearce et al. provided quantifiable evidence of the risk, demonstrating an increase in leukemia and brain tumors with higher radiation doses [6]. Another study has shown that even low doses of radiation during CT significantly increased the risk of hematological malignancies [9], suggesting that the risks of both solid and blood cancers were elevated. However, to date, few researchers have measured the specific doses of radiation or risk to each organ during CT.

Therefore, this study used cohort data from the National Health Insurance Service (NHIS) of South Korea to quantify the radiation doses attributable to pediatric CT and the relationships thereof with malignant tumor development. By analyzing a large cohort of pediatric patients, this study seeks to enhance awareness of and provide insight into the risks associated with radiation during CT and to encourage efforts that reduce radiation doses to children. Understanding the dose–response relationship between pediatric CT radiation exposure and cancer risk can guide future research in refining radiation safety protocols and contribute to clinical decision-making by promoting judicious CT use and alternative imaging modalities where feasible.

## Methods

### Data source

This nationwide observational cohort study was based on claims data from the NHIS, a mandatory national health insurance system that covers > 97% of the population and is operated by the Ministry of Health and Welfare in South Korea. The NHIS, managed by the Korean government, established a retrospective cohort database beginning in 2015; the database integrates the International Classification of Diseases Tenth Revision (ICD-10) codes, prescriptions, medical services, and costs paid out for inpatients and outpatients. All data are anonymized, collected regularly, and subjected to careful quality control. The study protocol was approved by the NHIS Institutional Review Board. The need for written informed consent from participants was waived because the NHIS data are anonymized. Our Institutional Review Board also approved the study protocol (approval no. 2022AS0014).

### Study cohorts

**Fig. 1:**
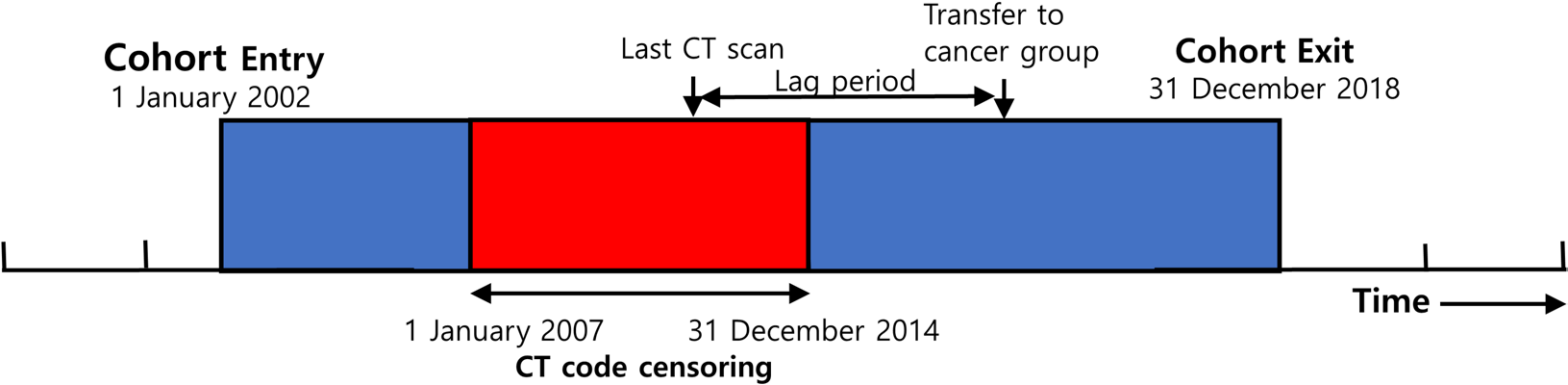
Schematic diagram of participant enrollment

**Fig. 2:**
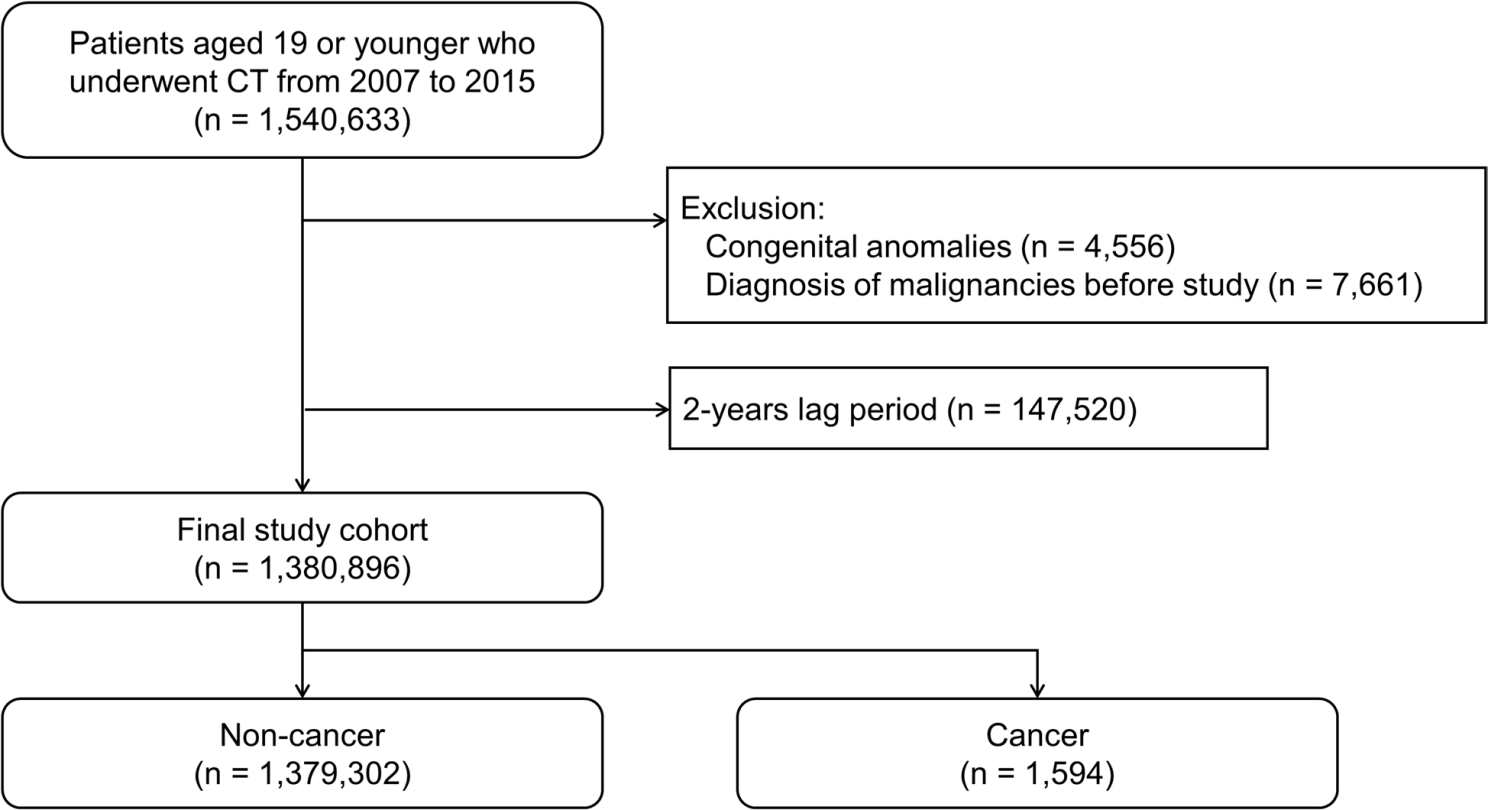
Flow diagram of the study

Participants entered the study on 1 January 2002 and exited on 31 December 2018, the date of death, or the date of cancer diagnosis (Fig. 1). Individuals < 20 years of age who underwent CT from 2007 to 2015 were selected for the exposed cohort using ICD-10 procedure codes for brain CT (HA441, HA451, HA461, HA471, HA481, HA801, HA805, HA809, HA813, and HA851), neck CT (HA443, HA453, HA463, HA473, HA483, and HA853), cervical spine CT (HA446, HA449, HA456, HA459, HA466, HA469, HA476, HA479, HA486, HA489, HA856, and HA859), chest CT (HA424, HA434, HA444, HA454, HA464, HA474, HA484, and HA834), abdominal CT (HA425, HA435, HA445, HA455, HA465, HA475, HA485, and HA835), upper-extremity CT (HA447, HA457, HA467, HA477, HA487, and HA857), and lower-extremity CT (HA448, HA458, HA468, HA478, HA488, and HA858). Subjects were excluded if they had congenital anomalies (V143, V144, V145, V146, V147, V148, V149, V150, V151, V154, V155, V156, V157, V158, V159, V160, V179, V180, V182, V183, V185, V186, V204, V205, V214, V215, V216, V217, V218, V220, V225, V226, V228, V229, V230, and V242) or were diagnosed with malignancies prior to the study entry date. We also excluded those exhibiting cancer development or death within the first 2 years of follow-up to minimize selection bias and screening effects (the lag period was thus 2 years). The 2-year lag period was selected based on previous large-scale cohort studies [10, 11, 27] that identified a minimum latency period of approximately 2 years for radiation-induced hematologic malignancies, particularly leukemia. This timeframe allows for sufficient delay to reduce reverse causation—where CT scans may have been performed in response to early cancer symptoms—while still capturing the short latency of pediatric hematologic cancers. For participants who had undergone multiple CT scans, the lag period was based on the date of the last CT examination. Figure 2 presents a diagram outlining the process of study participant selection, including inclusion and exclusion criteria.

### Definitions and classifications of cancers

Cancer diagnoses were based on ICD-10 codes [11] for mouth and pharynx cancer (C00–14), digestive system cancer (C15–26), respiratory system cancer (C3), bone cancer (C40–41), melanoma (C43–44), soft tissue cancer (C45–49), breast cancer (C50), female genital cancer (C51–58), male genital cancer (C60–63), urinary cancer (C64–68), brain cancer (C69–72), thyroid cancer (C73–75) unspecified cancer (C76–80), Hodgkin’s lymphoma (C81), other lymphoma (C82–83), other lymphoid cancer (C84-90), lymphoid leukemia (C91), other myeloid leukemia (C92–96), and myelodysplasia (D45–46, D47.1, D47.3), and (rare) incurable disease (V193). In South Korea, almost all cancer patients in the NHIS database are tagged with these ICD-10 codes, because the NHIS covers 95% of the medical expenses for cancer diagnoses and also offers a special support program for rare diseases. Consequently, the likelihood of missing any cancer patient was extremely low.

### Demographic and social variables

The NHIS database contains demographic and socioeconomic data including sex, age, household income, and place of residence. Age was divided into four groups: 0–4, 5–9, 10–14, and < 20 years (15–19). Low income was defined as an income < the 20th percentile. Place of residence was classified as urban or rural.

### Dosimetry

Organ doses were calculated using the National Cancer Institute dosimetry system for CT (NCICT) program developed at the National Cancer Institute (National Institutes of Health, USA) [12]. To obtain the necessary inputs, we used the NHIS billing codes for different CT scan types, the regulatory databases, and the literature to reconstruct dose estimates for all CT scans by year and patient age [13]. We established a library of Computed Tomography Dose Index based on multiple Korean CT dose surveys conducted between 2005 and 2019 and use the library for organ dose calculations [14–16]. Our calculations considered the body part scanned (head, neck, spine, chest, abdomen, upper and lower limbs), the year of the scan (2005–2019), and demographics such as gender (male, female) and age (0–20 years). The dosimetry data from 2005–2019 were included to ensure robust and comprehensive dose estimations, as they capture variations in CT radiation exposure over time. Although our study cohort consists of patients who underwent CT from 2007–2015, we utilized the full available range of dosimetry data to enhance the accuracy of organ dose calculations and account for changes in CT radiation dose protocols over the years. This approach, involving 3,780 potential combinations, allowed us to precisely calculate the radiation dose to each organ, thus considering brain, breast, thyroid, and lung exposures. Based on the detailed Korean CT dosimetric data, we generated 8,448 results by multiplying the 8 CT body parts, 44 CT effect types, and 24 cancer types (Table 1). Cumulative doses were calculated when multiple scans were conducted over time.

**Table 1:** The 8,448 Combinations Used for Precise CT Dose Calculations.

	**Body parts **	**CT effect types**	**Cancer types**
**Details **	Brain; Neck; Spine; Chest; Abdomen; Upper extremity; Lower extremity; Total	Brain; Shallow marrow; Active marrow; Adrenals; Breast; Colon; Esophagus; Eyeballs; Gall bladder; Heart wall; Kidney; Lens; Liver; Lungs; Muscle; Oral cavity; Ovaries; Pancreas; Pituitary gland; Prostate; Rectosigmoid; Salivary glands; Skin; Small intestine; Spinal cord; Spleen; Stomach wall; Testes; Thymus; Thyroid; Trachea; Urinary bladder; Uterus; Oral; Digestive; Respiratory tissue; Soft tissue; Female genitals; Male genitals; Urinary tissue; Brain; Thyroid; CTDIvol; Effective dose	Solid malignancies: Mouth and pharynx; Digestive tissue; Respiratory tissue; Bone; Melanoma; Soft tissue; Breast; Female genitals; Male genitals; Urinary system; Brain; Thyroid; Unspecified. Lymphoid and hematopoietic malignancies: Hodgkin's lymphoma; Other lymphomas; Other lymphoid cancers; Leukemias and myeloid [cancers?]; Leukemias; Lymphoid leukemia; Other myeloid cancers; Myelodysplasia; Total cancers
**Numbers**	8	44	24

CT, computed tomography; CTDIvol, volume computed tomography dose index.

*Total combinations: 8 × 44 × 24 = 8,448

### Statistical analysis

Categorical variables were analyzed using the chi-squared test, and continuous variables were compared using Student’s t-test. The IR was defined as the outcome per 100,000 PY divided by the total number of cancers. To evaluate the dose–response relationship, Cox proportional hazards regression was used to estimate hazard ratios (HRs) with 95% confidence intervals (CIs) for all cancer types based on radiation dose exposure. To minimize confounding effects, HRs were adjusted for key covariates, including age, sex, household income, and place of residence. We also calculated the HRs for cancer development according to quartiles of estimated organ doses. SAS software (ver. 9.4; SAS Institute, Cary, NC, USA) was used for all statistical analyses. A two-sided P-value < 0.05 was considered to indicate statistical significance.

## Results

In total, 1,540,633 children underwent CT from 2007 to 2015. We excluded 159,737 individuals based on the following criteria: congenital anomalies (n = 4,556), diagnosis of cancer before study entry (n = 7,661), and CT within the 2-year lag period (n = 147,520). Data from 1,380,896 children were included in the final analysis (Fig. 2).

### Baseline characteristics

Of the 1,380,896 children included in the analysis, 1,379,302 were classified into a non-cancer group and 1,594 into a cancer group. Notable disparities were observed in sex distribution and age between the two cohorts (P < 0.001 for both parameters). A predominance of males was observed in the non-cancer cohort (62.85%) compared to the cancer cohort (57.28%). The mean age at the time of CT was significantly lower in the cancer cohort (10.25 years) than the non-cancer cohort (12.06 years) (Table 2). During the study period, the frequencies of CT performed on different body parts and the associated volume computed tomography dose index (CTDIvol) values were calculated and are presented in Table 3.

**Table 2:** Baseline characteristics of the patients.

**Variables**	**Non-cancer patients**	**Cancer patients**	***p*-value**
	**(n = 1,379,302)**	**(n = 1,594)**	
Sex, n (%)			< 0.001
Male	866,863 (62.85)	913 (57.28)	
Female	512,439 (37.15)	681 (42.72)	
Age, years^†^	12.06 ± 4.7	10.25 ± 4.57	< 0.001
Age group, n (%)			< 0.001
0–4 years	119,827 (8.69)	218 (13.68)	
5–9 years	280,736 (20.35)	421 (26.41)	
10–14 years	441,490 (32.01)	629 (39.46)	
15–19 years	537,249 (38.95)	326 (20.45)	
Low income, n (%)	222,877 (16.16)	248 (15.56)	0.515
Place of residence, n (%)			0.533
Urban	600,685 (43.58)	707 (44.35)	
Rural	777,723 (56.42)	887 (55.65)	

The cancer group had a 2-year lag period.

^†^Mean ± standard deviation.

**Table 3:** Numbers of CT scans and CTDIvol examinations performed during the study period.

	Number of scans	CTDIvol (mGy)
Brain CT	719,974 (52.14)	48.24 ± 32.25
Neck CT	69,625 (5.04)	9.63 ± 4.73
Spine CT	94,910 (6.87)	9.95 ± 4.78
Chest CT	138,222 (10.01)	11.5 ± 7.98
Abdomen CT	434,327 (31.45)	10.9 ± 7.11
Upper-extremity CT	130,289 (9.44)	8.94 ± 4.41
Lower-extremity CT	136,098 (9.86)	9.2 ± 4.51

CT, computed tomography; CTDIvol, volume computed tomography dose index.CTDIvol values are presented as Mean ± Standard Deviation.

### Outcomes according to cancer type

Solid malignancies represented 60.23% of all cancer cases, and lymphoid and hematopoietic malignancies represented the remaining 39.77%. Among the solid malignancies, thyroid cancer (18.76%) was the most common, followed by brain cancer (12.8%) and female genital cancer (5.08%). Among the lymphoid and hematopoietic malignancies, leukemias accounted for the highest proportion, at 25.28%, with other myeloid tumors being the most frequent subtype at 17.13% (Table 4).

**Table 4:** Outcomes of CT-exposed pediatric patients by cancer type.

**Cancer type**	**Number of cancers**
	**(n = 1,594)**
**Solid malignancies, n (%)**	
Mouth and pharynx	38 (2.38)
Digestive system	74 (4.64)
Respiratory system	35 (2.2)
Bone	77 (4.83)
Melanoma	17 (1.07)
Soft tissue	57 (3.58)
Breast	4 (0.25)
Female genital	81 (5.08)
Male genital	27 (1.69)
Urinary	21 (1.32)
Brain	204 (12.8)
Thyroid	299 (18.76)
Unspecified	26 (1.63)
Total	960 (60.23)
**Lymphoid and hematopoietic malignancies, n (%)**	
Hodgkin's lymphoma	26 (1.63)
Other lymphomas	76 (4.77)
Other lymphoid cancers	81 (5.08)
Leukemias and myeloid cancers	451 (28.29)
Leukemias	403 (25.28)
Lymphoid leukemia	130 (8.16)
Other myeloid cancers	273 (17.13)
Myelodysplasia	48 (3.01)
Total	634 (39.77)

Based on the International Statistical Classification of Diseases and Related Health Problems, 10^th^ revision.

### Hazard ratios for cancer incidence relative to exposed organ doses

Cox’s proportional hazards regression analyses were performed to investigate the relationship between the exposed organ dose and cancer occurrence. After adjustment for age, sex, household income, and place of residence, a significant increase in the HR for cancer incidence was noted with each additional standard deviation of the exposed organ dose. The most pronounced associations with solid malignancies were those for mouth and pharynx cancer (HR 1.157, 95% CI: 1.045–1.282), digestive system cancer (HR: 1.285, 95% CI: 1.240–1.331), respiratory cancer (HR 1.165, 95% CI: 1.123–1.209), soft tissue cancer (HR 1.187, 95% CI: 1.150–1.225), urinary cancer (HR 1.385, 95% CI: 1.291–1.486), brain cancer (HR: 1.201, 95% CI: 1.177–1.225), and thyroid cancer (HR: 1.248, 95% CI: 1.218–1.278). In contrast, no significant associations were observed for bone cancer, melanoma, breast cancer, female genital cancer, or male genital cancer (*p* < 0.05). Among the lymphoid and hematopoietic malignancies, leukemias (HR: 1.074, 95% CI: 1.053–1.100), other myeloid tumors (HR: 1.087, 95% CI: 1.062–1.112), and myelodysplasia (HR: 1.096, 95% CI: 1.036–1.160) showed significant associations (Table 5, Additional File 1: Fig. S1). We compared the different HRs obtained using three lag periods (1, 2, and 5 years) to minimize the reverse causation of certain cancers (Additional File 1: Table S1).

**Table 5:** Hazard ratios for a one standard deviation increase in the exposed organ dose (mGy).

**Cancer type**	**Event**	**IR**^**†**^	**HR (95% CI)**	***p*-value**
**Solid malignancies**	960	13.89	1.169 (1.151–1.188)	< 0.001
Mouth and pharynx	38	0.55	1.157(1.045–1.282)	0.005
Digestive system	74	1.07	1.285 (1.240–1.331)	< 0.001
Respiratory system	35	0.51	1.165 (1.123–1.209)	< 0.001
Bone	77	1.11	1.078 (0.936–1.241)	0.299
Melanoma	17	0.25	0.871 (0.483–1.570)	0.647
Soft tissue	57	0.82	1.187(1.150–1.225)	< 0.001
Breast	4	0.06	1.136 (0.998–1.294)	0.054
Female genital	81	1.17	1.097 (0.980–1.188)	0.121
Male genital	27	0.39	0.951 (0.685–1.319)	0.763
Urinary	21	0.30	1.385 (1.291–1.486)	< 0.001
Brain	204	2.95	1.201 (1.177–1.225)	< 0.001
Thyroid	299	4.33	1.248 (1.218–1.278)	< 0.001
Unspecified	26	0.38	1.162 (1.049–1.287)	0.004
**Lymphoid and hematopoietic malignancies**	634	9.17	1.125 (1.094–1.158)	< 0.001
Hodgkin's lymphoma	26	0.38	1.074 (0.858–1.345)	0.531
Other lymphomas	76	1.10	1.047 (0.917–1.195)	0.500
Other lymphoid cancers	81	1.17	1.065 (0.972–1.166)	0.177
Leukemias and myeloid	451	6.53	1.076 (1.053–1.100)	< 0.001
Leukemias	403	5.83	1.074 (1.049–1.100)	< 0.001
Lymphoid leukemia	130	1.88	0.983 (0.852–1.133)	0.808
Other myeloid cancers	273	3.95	1.087 (1.062–1.112)	< 0.001
Myelodysplasia	48	0.69	1.096 (1.036–1.160)	0.001
**Total cancers**	1594	23.07	1.155 (1.139–1.171)	< 0.001

^†^IR was defined as events per 100,000 person-years.

Adjusted for age, sex, household income, and place of residence.

IR, incidence rate; HR, hazard ratio; CI, confidence interval.

### Cancer risk stratified by exposed organ dose quartiles

A clear dose–response relationship was apparent; the risk of cancer was higher in higher exposed organ dose quartiles (Table 6). A greater increase in risk was observed in the fourth quartile for solid malignancies (HR: 3.07, 95% CI: 2.50–3.77) than for lymphoid and hematopoietic malignancies (HR: 2.21, 95% CI: 1.76–2.77). Among solid malignancies, mouth and pharynx cancer (HR: 5.59, 95% CI: 1.66–18.87), digestive system cancer (HR: 8.62, 95% CI: 3.43–21.67), respiratory cancer (HR: 5.34, 955 CI: 1.19–23.90), female genital cancer (HR:3.92, 95% CI: 1.72–8.89), brain cancer (HR: 5.11, 95% CI: 3.18–8.22) and thyroid cancer (HR: 4.99, 95% CI: 3.41–7.31) exhibited notable increases in risk in the fourth quartile. Among lymphoid and hematopoietic malignancies, other lymphoid cancers (HR: 2.93, 95% CI: 1.38–6.19) and leukemia and myeloid cancers (HR: 2.01, 95% CI: 1.58–2.63) exhibited notable increases in risk in the fourth quartile.

**Table 6:** Hazard ratios for the development of cancers according to quartiles of exposed organ doses (mGy).

**Cancer type**	**HR (95% CI)**				
	**Q1**	**Q2**	**Q3**	**Q4**	**p-value**
**Solid malignancies**	1 (ref)	1.63 (1.30–2.05)	1.67 (1.34–2.09)	3.07 (2.50–3.77)	< 0.001
Mouth and pharynx	1 (ref)	1.64 (0.39–6.88)	2.95 (0.81–10.74)	5.59 (1.66–18.87)	0.007
Digestive system	1 (ref)	1.62 (0.54–4.85)	2.30 (0.81–6.52)	8.62 (3.43–21.67)	< 0.001
Respiratory system	1 (ref)	1.71 (0.31–9.34)	1.32 (0.22–7.92)	5.34 (1.19–23.90)	< 0.001
Bone	1 (ref)	1.19 (0.59–2.41)	0.92 (0.45–1.92)	1.90 (1.01–3.58)	0.064
Melanoma	1 (ref)	0.99 (0.20–4.93)	1.37 (0.33–5.78)	1.70 (0.42–6.86)	0.835
Soft tissue	1 (ref)	0.75 (0.33–1.70)	0.74 (0.33–1.66)	1.57 (0.79–3.10)	0.105
Breast	1 (ref)	-	1.97 (0.18–21.73)	0.93 (0.06–14.91)	0.916
Female genital	1 (ref)	3.65 (1.21–7.43)	2.24 (0.93–5.42)	3.92 (1.72–8.89)	0.007
Male genital	1 (ref)	2.17 (0.57–8.27)	2.69 (0.73–9.98)	2.18 (0.56–8.46)	0.531
Urinary	1 (ref)	0.64 (0.11–3.85)	1.52 (0.36–6.37)	3.35 (0.93–12.06)	0.063
Brain	1 (ref)	1.38 (0.78–2.43)	1.71 (0.99–2.94)	5.11 (3.18–8.22)	< 0.001
Thyroid	1 (ref)	1.30 (0.81–2.06)	1.10 (0.69–1.75)	4.99 (3.41–7.31)	< 0.001
Unspecified	1 (ref)	1.20 (0.32–4.47)	1.84 (0.55–6.13)	2.00 (0.61–6.51)	0.594
**Lymphoid and hematopoietic malignancies**	1 (ref)	1.27 (0.98–1.63)	1.19 (0.92–1.53)	2.21 (1.76–2.77)	< 0.001
Hodgkin's lymphoma	1 (ref)	1.26 (0.34–4.70)	1.12 (0.30–4.16)	2.58 (0.83–8.03)	0.212
Other lymphomas	1 (ref)	0.90 (0.40–2.00)	1.61 (0.82–3.15)	1.84 (0.95–3.58)	0.108
Other lymphoid cancers	1 (ref)	1.88 (0.84–4.22)	2.57 (1.20–5.48)	2.93 (1.38–6.19)	0.031
Leukemias and myeloid	1 (ref)	1.10 (0.82–1.48)	1.00 (0.74–1.34)	2.04 (1.58–2.64)	< 0.001
Leukemias	1 (ref)	1.05 (0.76–1.43)	0.97 (0.71–1.33)	2.01 (1.53–2.63)	< 0.001
Lymphoid leukemia	1 (ref)	1.05 (0.64–1.70)	0.65 (0.38–1.11)	1.20 (0.76–1.91)	0.120
Other myeloid cancers	1 (ref)	1.07 (0.71–1.60)	1.24 (0.85–1.83)	2.49 (1.76–3.50)	< 0.001
Myelodysplasia	1 (ref)	1.69 (0.67–4.30)	1.27 (0.48–3.34)	2.40 (1.01–5.73)	0.167
**Total cancers**	1 (ref)	1.46 (1.23–1.73)	1.44 (1.22–1.70)	2.66 (2.29–3.10)	< 0.001

Adjusted for age, sex, household income, and place of residence.

IR, incidence rate; HR, hazard ratio; CI, confidence interval; ref, reference.

### Cancer risk according to the body part subjected to CT

We analyzed the HRs for the organ dose during CT of various body parts according to cancer type. For brain cancer, the association between the organ dose of brain CT (HR: 1.186, 95% CI: 1.162–1.212) and cancer occurrence was most significant. For digestive system cancer, significant correlations were observed with both abdominal (HR: 1.310, 95% CI: 1.243–1.381) and chest (HR: 1.205, 95% CI: 1.048–1.387) CT, whereas mouth and pharynx cancers exhibited high correlations with neck CT (HR: 1.262, 95% CI: 1.142–1.396). For leukemias and myeloid cancer, significant associations with cancer occurrence were found for abdominal CT (HR 1.157, 95% CI: 1.059–1.265), chest CT (HR 1.127, 95% CI: 1.019–1.248), brain CT (HR: 1.080, 95% CI: 1.049–1.111), and spinal CT (HR: 1.247, 95% CI: 1.025–1.517) (Additional File 1: Table S2).

## Discussion

This large population-based cohort study utilizing the NHIS database of the Republic of Korea yielded real-world evidence of a dose–response relationship, indicating that the risk of cancer increases with the organ doses in pediatric patients undergoing CT. Notably, this association was more pronounced for certain types of solid cancers, including digestive system, respiratory, soft tissue, urinary, brain, and thyroid cancers, as well as for lymphoid and hematopoietic cancers, particularly leukemias and myelodysplasia. Our study demonstrated a clear dose–response relationship between radiation exposure and cancer development in children. It is important to note that the tissues of pediatric patients are generally more susceptible to the effects of radiation than those of adults given their rapid cell division and development [6]. This biological vulnerability implies that even low doses of radiation, if repeated and accumulated over time, can increase the risk of cancer [17]. Furthermore, higher doses of radiation have been shown to significantly elevate the potential for cancer development [18]. Therefore, clinicians should be aware that the use of CT for diagnosis of pediatric patients can increase radiation exposure and, consequently, the risk of cancer.

Previous studies have similarly identified a partial causal relationship between childhood CT exposure and the development of intracranial tumors. An Australian study investigating cancer incidence in 10.9 million people found that the overall incidence rate ratio (IRR) for brain cancer, based on a 1-year lag period, was 2.13 (CI 1.88–2.41) [9]. A German study reported a standardized incidence ratio (SIR) of 1.35 (95% CI: 0.54–2.78) for central nervous system tumors [19]. In Taiwan, the HR for all brain tumors was reported to be 2.56-fold higher in an exposed cohort (95% CI: 1.44–4.54, *P* < 0.01) compared to an unexposed cohort [20]. The frequency of CT showed a strong correlation with all brain tumors, with the HR increasing from 2.32 to 10.4 compared to the unexposed group (P = 0.0001) [20]. Our study supports these findings by demonstrating a significant association between CT exposure and brain cancer risk in a large-scale nationwide cohort. Moreover, while previous studies primarily examined CT exposure as a binary variable (exposed vs. unexposed), our study enhances this understanding by providing a dose–response analysis based on organ-level radiation dose estimations.

Similar to our findings, there is other evidence that radiation exposure in children poses a significant risk for the development of thyroid cancer. One study indicated that the risk of developing thyroid cancer was highest when patients were exposed to radiation at a younger age, with the risk decreasing as age increased and becoming lower around the age of 20 years [21]. Furthermore, even low-dose radiation exposure in childhood has been linked to an increased incidence of thyroid cancer [22, 23]. Our study supports these findings by demonstrating a dose–response relationship between radiation exposure and thyroid cancer risk, reinforcing the vulnerability of pediatric thyroid tissue to ionizing radiation. Unlike previous studies that primarily focused on radiation exposure without precise dosimetric assessment, our study utilizes organ-specific dose calculations, providing a more accurate quantification of radiation exposure and its impact on thyroid cancer development. Additionally, our findings indicate a significant association between organ doses and cancers of the digestive system and soft tissue. This may be attributable to the direct exposure of extensive regions such as the colon and soft tissues during CT, increasing the risk of malignancies in these organs [24].

One longitudinal analysis of 105,444 atomic bomb survivors from 1958 to 2009 revealed a strong and linear radiation dose–response relationship for urinary tract cancer, with an ERR/Gy of 1.4 (95% CI: 0.82–2.1) [25]. These findings are consistent with those of our study, which also revealed a significant association between urinary tract cancer and the radiation dose to that organ. Another study emphasized the association between diagnostic imaging involving ionizing radiation and an increased cancer risk, noting that radiation exposure triggering gastrointestinal malignancies predominantly involved abdominal and pelvic CT [26]. Our study also confirmed a significant relationship between the organ radiation dose and digestive system cancer, especially in cases undergoing abdominal and chest CT.

Studies of atomic bomb survivors have shown that most children developed leukemia as a result of radiation exposure. Excluding chronic lymphocytic leukemia, most types of leukemia can be induced by ionizing radiation, with a minimum latency period of about 2 years [27]. Therefore, in our study, we established a 2-year lag period to mitigate any bias arising from potential reverse causation, such as CT prompted by early cancer symptoms. Radiation from CT is absorbed by the red bone marrow, increasing the risk of leukemia in children [28]. This finding aligns with those of previous large cohort studies that reported a significant risk of pediatric leukemia associated with red bone marrow doses delivered during pediatric CT [9, 19]. Consistently, our study also found that as the organ doses increased, the risk of leukemia in children escalated.

An Intriguing aspect of our study was the tendency for solid tumors to exhibit higher HRs than hematological cancers. This contrasts with the findings of a study on Japanese atomic bomb survivors, who had a higher risk of hematological cancers than solid tumors [29]. This discrepancy may stem from the nature of radiation exposure: atomic bomb survivors experienced high-dose whole-body exposure, whereas our study focused on diagnostic CT radiation, where the radiation dose varies by body part. Given the inverse square law of radiation scattering [30], CT targeting specific body parts impacts those organs more significantly than other body parts, potentially “dispersing” the risk in cases with hematological cancers. In our study, the brain cancer risk was highest after brain CT, the digestive system cancer risk was highest after abdominal CT, and the mouth and pharynx cancer risk was highest after neck CT. Conversely, the leukemia and myeloid cancer risk was distributed across abdominal, chest, brain, and spine CT, resulting in a lower risk thereof compared to solid cancers.

Considering the established dose–response relationship between radiation exposure from CT and the increased risk of malignant tumors, especially in children, it is imperative that healthcare professionals establish strict protocols to minimize unnecessary radiation exposure. First, CT should be performed only when clinically necessary, and the lowest possible radiation dose that still affords adequate image quality permitting diagnosis should be used [6]. Second, priority should be given to alternative imaging methods that involve less or no radiation, such as ultrasound or magnetic resonance imaging, whenever these are clinically viable [9]. Third, healthcare providers must be well-informed about the radiation doses associated with CT, necessitating targeted education. A major factor contributing to the overuse of CT in children is a lack of awareness among medical professionals [19].

The present study had several notable strengths. To the best of our knowledge, it is the first study to investigate the risk of various cancer types using organ-level radiation doses in South Korea. By accurately measuring the radiation dose to each organ during CT that used the NCICT, we were able to provide more insight into the relationship between dose and the incidence of cancer in children. The precise dosimetry enhanced the reliability of our findings, shedding light on the direct impact of CT radiation on the pediatric cancer risk. Additionally, the data from the NHIS are representative of the Korean population, and the large sample size enhances the generalizability of our study results to this population. Although this study was based on South Korean data, the findings may be generalizable to other populations with similar CT utilization patterns. Given the universal radiosensitivity of pediatric tissues, the observed dose–response relationship is likely relevant globally, though differences in imaging guidelines and genetic factors should be considered. These findings underscore the importance of optimizing pediatric CT protocols to minimize radiation exposure while maintaining diagnostic accuracy. Furthermore, they provide a foundation for future research aimed at validating dose–response relationships in prospective cohorts and identifying individual susceptibility factors that may influence cancer risk.

However, this study also had several limitations First, the retrospective design may introduce selection bias, as imaging decisions and follow-up data were collected from past medical records rather than controlled prospective settings. We attempted to adjust for as many confounders as possible, but there may be other unaccounted-for confounding factors. Second, the reliance on administrative data limits the availability of detailed clinical information, such as the exact indications for CT scans or individual risk factors, which may influence cancer outcomes. Although the decision to perform CT was made by clinicians based on medical judgment, we cannot assume that all cancers observed during the follow-up period were related to CT exposure; there is a possibility of reverse causation [31]. However, we designed the study with a 2-year lag period to minimize this possibility, and a similar trend was observed even with a maximum 5-year lag period (Additional File 1: Table S1). Third, although the biological mechanisms of radiation-induced malignancy are universal, the generalizability of our findings to non-Korean populations remains limited. Ethnic and genetic variability may influence individual susceptibility to cancer, and significant differences exist in CT utilization patterns, diagnostic criteria, and healthcare access across countries. These factors may alter both exposure and detection rates, and therefore, our findings should be extrapolated to other populations with caution. Further well-designed studies are needed to overcome these limitations.

## Conclusions

This study revealed a significant association between increased radiation doses during CT and a higher risk of various cancers in pediatric patients. These findings underscore the importance of employing CT judiciously in children, given the potential long-term risks. While CT remains an invaluable diagnostic tool, even at current diagnostic dose levels, careful risk–benefit analysis is warranted—especially in pediatric care.

To mitigate unnecessary exposure, healthcare providers should prioritize the implementation of pediatric-specific low-dose CT protocols and promote the use of alternative imaging modalities, such as ultrasound or MRI, where appropriate. Moving forward, the integration of standardized, evidence-based low-dose protocols across institutions is essential to balance diagnostic utility with patient safety.

Additionally, effective and transparent communication with parents and guardians regarding potential risks, benefits, and imaging alternatives is critical. Developing targeted educational materials and decision aids can further support shared decision-making and informed consent, fostering trust in clinical care.

## Supplementary Information

Additional file 1: Fig. S1 Forest plot of the hazard ratios and 95% confidence intervals for different cancer types. HR, hazard ratio; CI, confidence interval. Table S1. Hazard ratios according to the lag period with one standard deviation increase in the CTDIvol. Table S2. Hazard ratios for exposed organ doses (mGy) to body parts subjected to CT according to cancer type.

## Data Availability

In accordance with Korean law, the study authors are not permitted to transfer any data files to a third party. However, data are available from the Korea National Health Insurance Sharing Service Institutional Data Access/Ethics Committee (https://nhiss.nhis.or.kr/bd/ay/bdaya001iv.do) for researchers who meet the criteria for access to confidential data. The authors had no special access privileges to the data and other researchers will be able to access the data in the same manner as the authors.

## Abbreviations

CI: Confidence interval
CT: Computed tomography
CTDIvol: Volume computed tomography dose index
HR: Hazard ratio
ICD-10: International Classification of Diseases, Tenth Revision
IR: Incidence rate
IRR: Incidence rate ratio
NCICT: National Cancer Institute dosimetry system for computed tomography
NHIS: National Health Insurance Service
PY: Person-years
SIR: Standardized incidence ratio
C00–14: Mouth and pharynx cancer
C15–26: Digestive system cancer
C3: Respiratory system cancer
C40–41: Bone cancer
C43–44: Melanoma
C45–49: Soft tissue cancer
C50: Breast cancer
C51–58: Female genital cancer
C60–63: Male genital cancer
C64–68: Urinary cancer
C69–72: Brain cancer
C73–75: Thyroid cancer
C76–80: Unspecified cancer
C81: Hodgkin’s lymphoma
C82–83: Other lymphoma
C84-90: Other lymphoid cancer
C91: Lymphoid leukemia
C92–96: Other myeloid leukemia
D45–46, D47.1, D47.3: Myelodysplasia
V193: Rare incurable disease
