# Supplementary material for: Pediatric computed tomography scan and subsequent risk of malignancy: a nationwide population-based cohort study in Korea using National Cancer Institute dosimetry system for computed tomography (NCICT)
Source: BMC Med. 2025 Jul 1;23:355. doi: 10.1186/s12916-025-04235-3 (PMC12211530; doi:10.1186/s12916-025-04235-3)
Supplement: Supplementary file 1 — Additional file 1: Fig. S1 Forest plot of the hazard ratios and 95% confidence intervals for different cancer types. HR, hazard ratio; CI, confidence interval. Table S1. Hazard ratios according to the lag period with one standard deviation increase in the CTDIvol. Table S2. Hazard ratios for exposed organ doses (mGy) to body parts subjected to CT according to cancer type. [file 12916_2025_4235_MOESM1_ESM.docx]

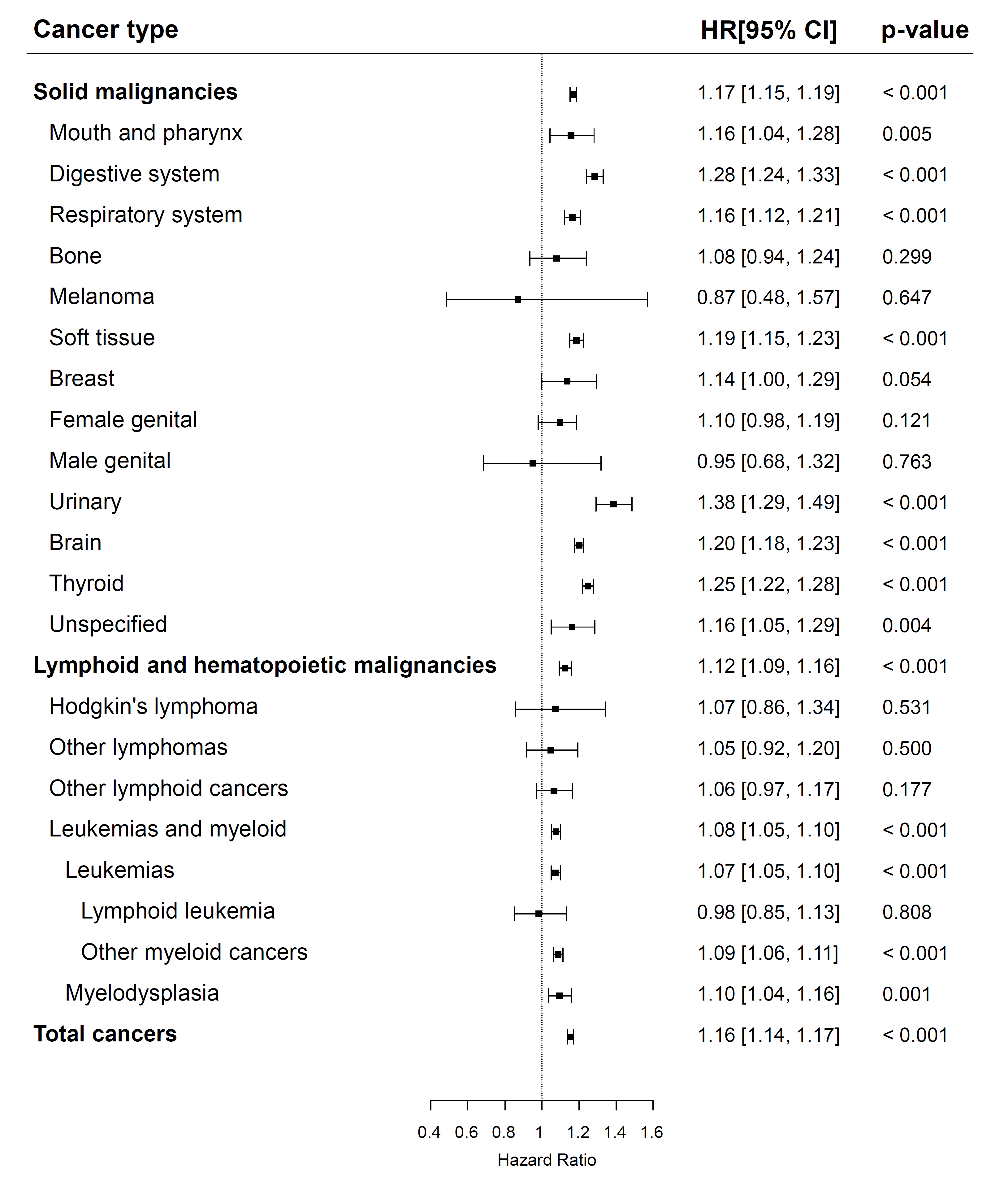


**Figure S1. Forest plot of the hazard ratios and 95% confidence intervals for different cancer types. HR, hazard ratio; CI, confidence interval.**

**Table S1. Hazard ratios according to the lag period with one standard deviation increase in the CTDIvol.**

|  | **1-year lag** | **2-year lag** | **5-year lag** |
| --- | --- | --- | --- |
| **Cancer type** | **HR (95% CI)** | **HR (95% CI)** | **HR (95% CI)** |
| **Solid malignancies** | 1.167(1.151,1.184) | 1.169(1.151,1.188) | 1.178(1.152,1.204) |
| Mouth and pharynx | 1.13(1.012,1.262) | 1.135(1.011,1.273) | 1.132(0.963,1.329) |
| Digestive system | 1.145(1.07,1.226) | 1.152(1.073,1.237) | 1.158(1.043,1.284) |
| Respiratory system | 1.134(1.011,1.272) | 1.142(1.011,1.289) | 1.15(0.986,1.341) |
| Bone | 1.091(0.98,1.215) | 1.078(0.936,1.241) | 1.085(0.906,1.299) |
| Melanoma | 1.109(0.91,1.351) | 1.062(0.754,1.496) | 1.02(0.529,1.966) |
| Soft tissue | 1.185(1.136,1.236) | 1.196(1.147,1.248) | 1.206(1.147,1.268) |
| Breast | 0.754(0.184,3.094) | 0.752(0.18,3.135) | - |
| Female genital | 1.126(1.032,1.228) | 1.133(1.036,1.24) | 1.159(1.032,1.301) |
| Male genital | 1.143(1.016,1.287) | 1.154(1.029,1.294) | 1.138(0.899,1.441) |
| Urinary | 1.177(1.09,1.271) | 1.176(1.073,1.288) | 1.209(1.101,1.328) |
| Brain | 1.201(1.181,1.221) | 1.2(1.177,1.225) | 1.206(1.171,1.242) |
| Thyroid | 1.106(1.048,1.169) | 1.117(1.057,1.18) | 1.138(1.066,1.216) |
| Unspecified | 1.155(1.042,1.28) | 1.162(1.049,1.287) | 0.712(0.255,1.986) |
| **Lymphoid and hematopoietic malignancies** | 1.120(1.09,1.151) | 1.125(1.094,1.158) | 1.124(1.077,1.174) |
| Hodgkin's lymphoma | 1.074(0.853,1.351) | 1.102(0.898,1.353) | 0.832(0.404,1.714) |
| Other lymphomas | 1.085(0.965,1.22) | 1.099(0.979,1.233) | 1.113(0.979,1.265) |
| Other lymphoid cancers | 1.094(0.983,1.218) | 1.091(0.963,1.236) | 1.064(0.84,1.348) |
| Leukemias and myeloid | 1.127(1.095,1.16) | 1.132(1.098,1.166) | 1.135(1.085,1.187) |
| Leukemias | 1.126(1.092,1.161) | 1.129(1.093,1.167) | 1.135(1.083,1.19) |
| Lymphoid leukemia | 1.001(0.86,1.165) | 1.018(0.875,1.185) | 1.074(0.925,1.247) |
| Other myeloid cancers | 1.145(1.111,1.179) | 1.149(1.113,1.186) | 1.153(1.098,1.211) |
| Myelodysplasia | 1.139(1.043,1.243) | 1.149(1.055,1.251) | 1.129(0.961,1.325) |
| **Total cancers** | 1.153(1.139,1.167) | 1.155(1.139,1.171) | 1.161(1.139,1.184) |
| Adjusted for age, sex, household income, and place of residence.  CTDIvol, volume computed tomography dose index; HR, hazard ratio; CI, confidence interval. | | | |

**Table S2. Hazard ratios for exposed organ doses (mGy) to body parts subjected to CT according to cancer type.**

| **Cancer type** | **Event** | **IR^†^** | **HR (95% CI)** | **p-value** |
| --- | --- | --- | --- | --- |
| **Solid malignancies** |  |  |  |  |
| **Brain ca.** (All CTs) | 204 | 2.95 | 1.201 (1.177–1.225) | < 0.001 |
| + Abdomen CT | 47 | 2.36 | 0.947 (0.702–1.278) | 0.723 |
| + Chest CT | 16 | 2.87 | 1.040 (0.689–1.569) | 0.852 |
| + Neck CT | 11 | 3.12 | 1.198 (0.863–1.662) | 0.282 |
| + Brain CT | 167 | 3.97 | 1.186 (1.162–1.212) | < 0.001 |
| + Spine CT | 13 | 3.79 | 1.081 (0.713–1.641) | 0.713 |
| + Upper-extremity CT | 11 | 1.66 | 1.091 (0.625–1.904) | 0.758 |
| + Lower-extremity CT | 20 | 3.01 | 0.832 (0.511–1.356) | 0.461 |
| **Digestive ca.** (All CTs) | 74 | 1.07 | 1.285 (1.240–1.331) | < 0.001 |
| + Abdomen CT | 54 | 2.72 | 1.310 (1.243–1.381) | < 0.001 |
| + Chest CT | 17 | 3.05 | 1.205 (1.048–1.387) | 0.009 |
| + Neck CT | 6 | 1.75 | 1.239 (0.882–1.740) | 0.217 |
| + Brain CT | 36 | 0.85 | 0.869 (0.505–1.495) | 0.613 |
| + Spine CT | 6 | 1.75 | 0.485 (0.086–2.741) | 0.413 |
| + Upper-extremity CT | 9 | 1.36 | 1.189 (0.911–1.551) | 0.202 |
| + Lower-extremity CT | 13 | 1.96 | 0.942 (0.539–1.645) | 0.833 |
| **Mouth and pharynx ca.** (ALL CTs) | 38 | 0.55 | 1.157(1.045–1.282) | 0.005 |
| + Abdomen CT | 10 | 0.50 | 0.666 (0.235–1.889) | 0.445 |
| + Chest CT | 5 | 0.90 | 0.376 (0.038–3.762) | 0.405 |
| + Neck CT | 27 | 7.88 | 1.262(1.142–1.396) | < 0.001 |
| + Brain CT | 22 | 0.52 | 1.073 (0.827–1.391) | 0.595 |
| + Spine CT | 3 | 0.87 | 0.301 (0.013–6.850) | 0.451 |
| + Upper-extremity CT | 3 | 0.45 | 1.025 (0.378–2.779) | 0.962 |
| + Lower-extremity CT | 2 | 0.30 | 0.993 (0.290–3.400) | 0.992 |
| **Lymphoid and hematopoietic malignancies** |  |  |  |  |
| **Leukemias and myeloid cancers** (All CTs) | 451 | 6.53 | 1.076 (1.053–1.100) | < 0.001 |
| + Abdominal CT | 179 | 9.00 | 1.157 (1.059–1.265) | 0.001 |
| + Chest CT | 84 | 15.08 | 1.127 (1.019–1.248) | 0.021 |
| + Neck CT | 38 | 11.09 | 1.160 (0.995–1.352) | 0.058 |
| + Brain CT | 291 | 6.91 | 1.080 (1.049–1.111) | < 0.001 |
| + Spine CT | 31 | 9.04 | 1.247 (1.025–1.517) | 0.027 |
| + Upper-extremity CT | 39 | 5.90 | 1.155 (0.924–1.443) | 0.207 |
| + Lower-extremity CT | 35 | 5.27 | 1.099 (0.844–1.431) | 0.484 |
| ^†^IR defined as events per 100,000 person-years.  Adjusted for age, sex, household income, and place of residence.  CTDIvol, volume computed tomography dose index; IR, incidence rate; HR, hazard ratio; CI, confidence interval. | | | | |
